# Giant Sublingual, Submental, and Lingual Dermoid Cyst Restricting Tongue Movement Undiagnosed for Several Years

**DOI:** 10.3390/diseases12050091

**Published:** 2024-05-06

**Authors:** Jakub Bargiel, Michał Gontarz, Krzysztof Gąsiorowski, Tomasz Marecik, Paweł Szczurowski, Jan Zapała, Grażyna Wyszyńska-Pawelec

**Affiliations:** Department of Cranio-Maxillofacial Surgery, Jagiellonian University Medical College, 30-688 Cracow, Poland; michal.gontarz@uj.edu.pl (M.G.); krzysztof.gasiorowski@uj.edu.pl (K.G.); tomasz.marecik@uj.edu.pl (T.M.); pawel.szczurowski@uj.edu.pl (P.S.); jan.zapala@uj.edu.pl (J.Z.); grazyna.wyszynska-pawelec@uj.edu.pl (G.W.-P.)

**Keywords:** dermoid cyst, epidermoid cyst, sublingual, submental

## Abstract

(1) Background: Dermoid cysts occurring in the sublingual space are uncommon, typically manifesting as painless, gradually enlarging masses, usually not exceeding 3 cm in diameter. These cysts can resemble various conditions due to their clinical presentation, with a relatively low occurrence rate in the oral cavity, accounting for about 1.6% of all dermoid cysts. (2) Methods: We present the case of a 17-year-old female with a giant dermoid cyst involving the submental, sublingual, and lingual areas, undiagnosed for several years. Diagnosis was achieved through MRI and fine-needle aspiration, leading to the decision for surgical removal through a cervical approach. (3) Results: The healing process was uneventful. From the first day post-surgery, the patient began myofunctional therapy, successfully regaining proper tongue functions. Throughout a 24-month follow-up, the patient remained symptom-free. (4) Conclusions: A cervical approach can successfully treat giant oral dermoid cysts involving submental, sublingual, and lingual spaces. Tongue function can be successfully regained through myofunctional therapy after surgical treatment.

## 1. Introduction

Three types of cysts with similar symptoms but a different histology and content can be found in the sublingual space: the epidermal cyst, dermoid cyst, and teratoid cyst [1]. Dermoid cysts and the more frequently occurring epidermoid cysts are observed in nearly 60% of cases of Gardner’s syndrome and familial polyposis. These cysts are typically located on the face, scalp, and extremities [2].

Dermoid cysts are rare in the head and neck region, and it is exceptionally uncommon for them to expand to significant sizes and spread into surrounding anatomical regions, including the submental, submandibular, lingual, and parapharyngeal spaces [3]. These cysts are characterized by cavities lined with epithelium and filled with skin appendages, including hairs, hair follicles, sebaceous glands, sweat glands, and diverse elements of the underlying connective tissue. In the sublingual region, dermoid cysts typically present as a smooth, dough-like mass that pushes the tongue towards the palate. In contrast, when these cysts extend inferiorly, they often appear as a pendulous mass beneath the mandible [4].

Patients with a dermoid cyst of the sublingual area typically present with symptoms that may prompt them to seek medical attention. The most common symptom is a noticeable mass or swelling in the floor of the mouth, which can gradually increase in size over time. This swelling is usually painless, but its presence can lead to discomfort or difficulty with oral functions such as speaking, chewing, and swallowing. In some cases, the mass may interfere with the movement of the tongue or cause a sensation of fullness underneath the chin or throat. Patients might also report a feeling of obstruction that affects their breathing or causes snoring. The onset of these symptoms often leads individuals to consult healthcare professionals for diagnosis and management early.

Diagnostic imaging plays a crucial role in identifying and managing dermoid cysts located in the mouth’s floor. Imaging techniques are essential for accurately diagnosing these cysts and determining their size, extent, and relationship to surrounding anatomical structures, which is critical to planning the appropriate surgical intervention. Imaging modalities such as computed tomography (CT), ultrasonography (US), and magnetic resonance imaging (MRI) can be used to diagnose mass lesions situated in the sublingual or submental space.

We present an exceptional case of a giant sublingual cyst extending into the submental and lingual areas, significantly limiting tongue mobility, which remained undiagnosed for several years. 

## 2. Case Report

A 17-year-old female patient was referred to our institution by her dentist due to a large mass in the sublingual area, elevating the tongue and restricting its movement. This lesion was discovered incidentally during a routine dental appointment, as neither the patient nor her mother had noticed anything unusual. Remarkably, the patient did not experience any issues related to eating, swallowing, or breathing in the past. However, it is interesting to note that the patient had complained of experiencing a lisp and snoring for several years. Initially, a painless swelling was observed in the submental region. Nevertheless, it was somewhat overlooked, potentially due to the patient’s first-degree obesity, as indicated by a Body Mass Index (BMI) of 31. There was no history of any surgical interventions or trauma to the oral cavity or the neck region in the patient’s medical history. 

**Figure 1:**
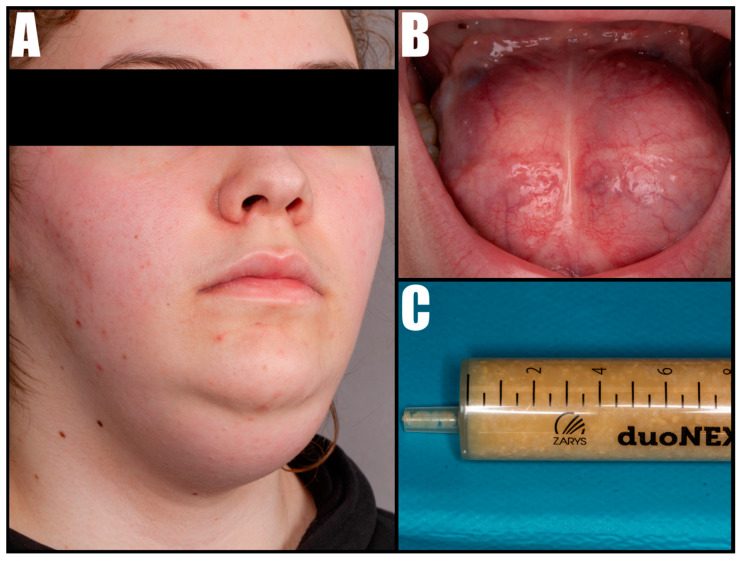
External view of the submental mass (**A**) and intraoral view of the cystic lesion involving the tongue (**B**). A fine-needle aspiration biopsy showed dense fluid with keratinous masses (**C**).

Upon clinical examination, a well-defined, firm, and painless mass was observed in the submental area, with an elevation of the mouth’s floor and the tongue’s ventral surface, resulting in an upward displacement. Additionally, a symmetrical mass located underneath the tongue was identified, characterized by a yellow, dense content visible through the thin mucous membrane covering the floor of the mouth (Figure 1). Tests performed to evaluate the motor function of the tongue revealed a restriction in its movement. The patient was unable to protrude their tongue. Both upward and lateral movements of the tongue were significantly restricted, highlighting the mass’s impact on oral functions.

**Figure 2:**
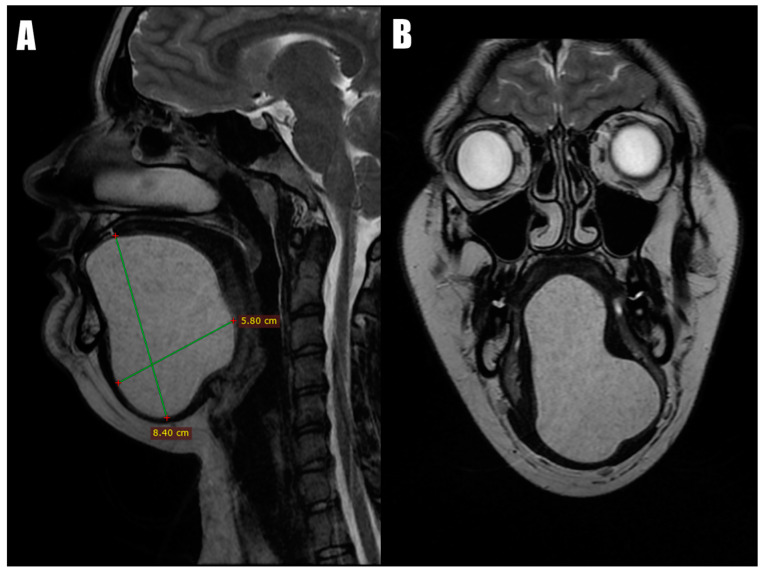
MRI scans: the coronal view (**A**) and the axial view (**B**) demonstrate that the submental and sublingual dermoid cyst extended superiorly, dividing intrinsic tongue muscles.

Magnetic Resonance Imaging (MRI) revealed a well-defined T1 hypointense cystic lesion and a T2 hyperintense cystic lesion located above the mylohyoid and geniohyoid muscles (Figure 2). The base of the tongue was slightly displaced, leading to a narrowing of the upper airway passage. The cystic lesion extended from the submental area to the floor of the mouth and superiorly into the tongue, measuring 5.8 × 8.4 cm at its largest dimensions. A fine-needle aspiration cytology (FNAC) revealed a dense, mud-like content with keratin and anucleated squamous cells without any signs of inflammation, suggesting the diagnosis of a dermoid cyst. Following this diagnosis, the patient was promptly scheduled for surgical intervention.

**Figure 3:**
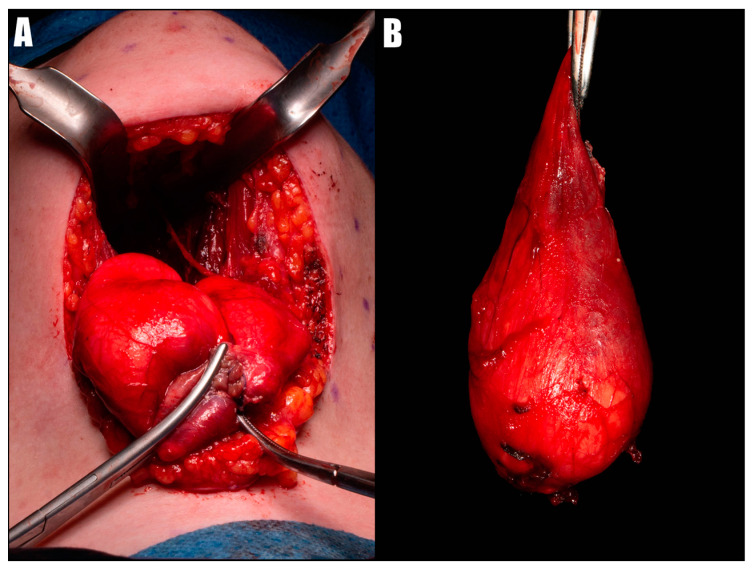
Intraoperative view of dermoid cyst removal after partial decompression (**A**) and cyst completely enucleated (**B**).

A cervical approach was chosen to reduce the risk of damaging the tongue muscles, lingual nerves, sublingual glands, and salivary ducts and to minimize the risk of oral bacterial infection. The patient expressed no concern regarding the potential for unaesthetic scar formation. The surgical procedure was carried out under general anesthesia with nasotracheal intubation (NTI), allowing for the administration of anesthetic gases without restricting access to the intraoral anatomy. One dose of antibiotic prophylaxis was administered before the operation. An incision of approximately 4 cm was made following the neck crease and the curvature of the mandible’s lower border. The mylohyoid and geniohyoid muscles were identified and divided by blunt dissection, carefully avoiding the submental vessels. Retractors were then used to gently pull the tissues laterally, facilitating the identification of the cyst capsule. The thick-walled cyst extended beyond the region of the hyoid bone without connecting to it, filling the space of the floor of the mouth, the body, and the base of the tongue and adjacent to the anterior pharyngeal wall. The cyst was delicately separated from surrounding tissues using blunt-ended scissors, and its capsule was then opened through a small incision. This allowed for partial cyst draining, reducing its size to lower the risk of accidental rupture and to facilitate the removal process. After suturing the capsule, the cyst was removed entirely, with the oral mucosa left intact (Figure 3). Bleeding throughout the surgical procedure was minimal. There was no need for any vessel ligation. A Redon drainage tube was left in the wound for one day to reduce the risk of hematoma formation. The patient was discharged from the hospital on the second day after the surgery. 

**Figure 4:**
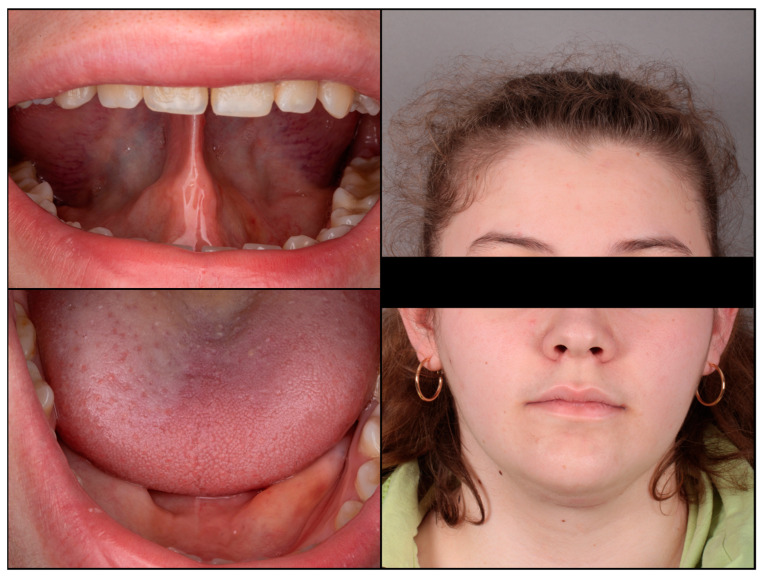
Patient symptom-free six months after the surgery.

The patient’s recovery was uneventful. The sutures were removed after seven days. Subsequently, a histopathological analysis of the removed cystic tumor confirmed the initial diagnosis of a dermoid cyst. Regular follow-up visits, including ultrasound examinations, were scheduled every six months at an outpatient clinic to monitor the patient’s progress and ensure no recurrence. No cyst recurrence was observed at the 24-month follow-up (Figure 4). The patient expressed satisfaction with the aesthetic appearance of the scar.

Despite the cyst involving a large portion of the tongue, the patient regained the ability to move the tongue in every direction immediately after the operation. This rapid recovery allowed the initiation of myofunctional therapy with a speech therapist starting the first day after surgery, focusing on restoring the tongue’s strength, endurance, and functionality. During the first week, the patient performed only gentle exercises, lifting the tongue to the incisive papilla and reaching the tongue to the upper premolars with the mouth open. Other exercises in all directions, including lingual to palatal suction and proper speech therapy, were introduced during the second week. As a result, the patient experienced significant improvement in tongue function, including improved swallowing, sleeping without snoring, and speaking without lisping.

## 3. Discussion

Epidermoid and dermoid cysts in the oral cavity are rare findings, making up less than 0.01% of all cysts in the oral cavity [5]. Dermoid cysts are typically observed in childhood and may be found in various locations. These cysts commonly appear in regions where embryonic components merge, such as the sacral area and ovaries. They are less frequent in the head and neck region, accounting for less than 7% of cases. Within this region, they are often located in the periorbital area, especially near the lateral aspect of the eyebrows. The floor of the mouth is the second most frequent location in the head and neck region, though it hosts only 1.6% of all dermoid cysts [5]. They may also be found on the lip, tongue, cheek, and other locations within the oral mucosa [6].

Two main theories have been proposed to explain the pathogenesis of dermoid cysts. The first, known as the acquired implantation theory, suggests that dermoid cysts may form as a result of the traumatic insertion of epithelial cells into deeper tissues or as a result of the occlusion of sebaceous glands [7]. Such cysts typically manifest after certain types of trauma or surgery, suggesting a direct link to external injury. The second and more widely accepted theory is that of congenital inclusion cysts. This concept indicates that dermoid cysts develop due to the entrapment of epithelial cells during the embryonic phase, specifically during the fusion of the first and second pharyngeal arches in the midline, which occurs between the third and fourth weeks of gestation. This theory supports the idea that dermoid cysts have a developmental origin linked to the complex process of human embryogenesis [8].

Clinically, dermoid cysts usually present as a painless, slow-growing mass in the sublingual, submandibular, and sublingual regions. An oral dermoid cyst localized sublingually begins in the midline but may expand laterally and inferiorly, reaching a large size before presenting clinical symptoms. However, there may be a sudden increase in the size of these lesions, and this may be due to the onset of puberty when there is an increase in sebum secretion from the sebaceous glands, or a secondary infection of the cyst contents may cause it. The location of the cyst relative to the geniohyoid and mylohyoid muscles affects its external presentation. Cysts above these muscles often result in sublingual swelling and may cause tongue displacement. On the other hand, cysts located below these muscles are associated with submental swelling or a fuller appearance, which can lead to the visual impression of a double chin. This can sometimes result in a misdiagnosis, as seen in the case presented. Dermoid cysts are usually found along the midline, though a lateral position involving the submandibular space can also be encountered [9]. When the sublingual and lingual spaces are occupied, symptoms similar to those of ankyloglossia, including difficulties with swallowing, chewing, breathing, and speaking, may occur [10,11].

While CT scans are a viable option, an MRI and ultrasound are preferred because of their superior soft tissue resolution, which offers a clearer view of a lesion’s internal structure. The MRI is considered the gold standard due to its exceptional soft tissue contrast, allowing for an accurate definition of the cyst’s borders and its relationship to adjacent tissues. In this specific case, the diagnosis was confirmed through MRI imaging.

Enucleation surgery is the primary and most effective treatment method for dermoid cysts. The selection of the surgical approach is critically influenced by the cyst’s anatomical location, particularly its relationship to the geniohyoid and mylohyoid muscles, as well as the cyst’s overall size, for larger cysts, especially those that span at least two anatomical spaces, a cervical approach is preferred. This external approach allows for better access and control in removing larger lesions, minimizing the cyst rupture risk, and ensuring thorough cystic content removal. Smaller lesions located superficial to the geniohyoid and mylohyoid muscles can often be effectively treated using an intraoral approach. This method is less invasive and can benefit in terms of recovery time and cosmetic outcomes by avoiding external scarring [12]. The choice between these surgical approaches is made after a detailed assessment of the cyst through diagnostic imaging.

The differential diagnosis for dermoid cysts located in the floor of the mouth includes a variety of conditions: a ranula (including plunging ranula), obstruction of Wharton’s duct, thyroglossal duct cyst, cystic hygroma, branchial cleft cyst, acute infection or cellulitis of the mouth floor, infections of the submandibular and sublingual glands, vascular and lymphatic anomalies, benign and malignant tumors of the mouth floor, and adjacent salivary glands, as well as hypertrophy of fatty tissue in the submental area [13,14,15,16,17]. 

Dermoid cysts can lead to complications such as inflammation with abscess formation, dysphagia, dystonia, disorders of swallowing, chewing, or vocal function, airway obstruction, or malignant transformation [18]. These complications are primarily related to the size of the cyst or infection of its contents as a result of trauma or surgical interventions. Giant sublingual cysts protrude into the tongue and submental area, creating the appearance of a “double chin,” as in our case. A malignant transformation in dermoid cysts is a rare complication in approximately 5% of cases [18,19]. 

## 4. Conclusions

Dermoid cysts found in the sublingual, lingual, and submental areas are uncommon developmental occurrences. Due to their rarity and non-specific clinical symptoms, they are frequently misidentified. Timely identification and intervention are essential to avert the risk of inflammation with acute airway obstruction. Enucleation is the preferred treatment method, with the surgical approach being determined by the size and location of the cyst in relation to the geniohyoid and mylohyoid muscles. 

Giant sublingual dermoid cysts can impair the tongue’s oral functions, such as eating, swallowing, speaking, and breathing. Merely removing the cyst, which restricts the tongue’s mobility, does not instantly restore these functions. Recovery is a gradual process that requires time and specific exercises. Myofunctional therapy is a safe option following the surgical removal of a giant dermoid cyst and should be recommended for other conditions affecting the tongue.

## Data Availability

Restrictions apply to the availability of these data. The data were obtained from patients treated at the Department of Cranio-Maxillofacial Surgery, Cracow, Poland, and cannot be shared in accordance with the General Data Protection Regulation (EU) 2016/679.
